# Diagnostic Dilemma in the Treatment of a Fatal Case of Bloody Diarrhea

**DOI:** 10.1177/2324709616638698

**Published:** 2016-03-17

**Authors:** Sidharth Mahapatra, Sara A. Michie, Karl Sylvester, David Cornfield

**Affiliations:** 1 Division of Critical Care, Department of Pediatrics, University of Nebraska Medical Center, Omaha, NE, USA; 2 Department of Pathology, Lucile Packard Children’s Hospital Stanford, Palo Alto, CA, USA; 3 Department of Surgery, Lucile Packard Children’s Hospital Stanford, Palo Alto, CA, USA; 4 Department of Pediatrics, Lucile Packard Children’s Hospital Stanford, Palo Alto, CA, USA

**Keywords:** diarrhea, food protein–induced enterocolitis syndrome, intussusception, septic shock

## Abstract

Although diarrhea is the most commonly reported pediatric illness in the United States, mortality is usually a rare and unexpected event. We report the case of a healthy 13-month-old male that succumbed to a diarrheal illness of unclear etiology. Presenting signs included frequent nonbloody stools that progressed to frankly bloody stools over 72 hours. Associated symptoms included fever, tenesmus, relief with stool passage, and significant fatigue. On examination, the patient appeared tired and lay with legs curled toward his chest. The abdominal exam was remarkable for hypoactive bowel sounds, diffuse tenderness to palpation without guarding or rebound pain, and intermittent prolapse of rectal tissue. Abdominal plain films demonstrated a paucity of bowel gas, especially in the rectum; and ultrasound revealed thickening of bowel loops in the left lower quadrant. Abdominal computed tomography scan showed decreased enhancement of the mucosa of the rectosigmoid colon. The patient deteriorated rapidly with cardiorespiratory arrest occurring 48 hours after admission. Despite a protracted effort at cardiopulmonary resuscitation, perfusing heart rate or rhythm could not be reestablished. Autopsy revealed infarction and necrosis of the rectosigmoid colon with invasive gram-negative bacilli. Here we present his perplexing case, diagnostic evaluations, and suggest a unifying diagnosis.

## Introduction

Diarrheal illnesses are the leading cause of visits to the pediatrician. Infection is the most common cause of diarrhea in the pediatric age group, although allergic etiologies are becoming more prevalent. Infectious gastroenteritis accounts for more than 1.5 million outpatient visits and 200 000 hospitalizations annually.^1,2^ While viruses like rotavirus and adenovirus account for the majority of cases, common bacterial causes include *Escherichia coli, Salmonella*, and *Shigella*. Parasites, such as *Giardia intestinalis* and *Cryptosporidium*, represent less than 5% of cases.^3^ Fecal-oral contamination is the most common route for contraction of the illness; hence, daycare centers are notorious sources for diarrheal illnesses in infants and toddlers.^4^ Exposure to unpasteurized milk and meats increases the risk for contraction of *E coli, Salmonella*, and *Listeria*.

Worldwide, infectious diarrhea is the second leading cause of death in children under 5 years of age.^5^ Oral rehydration therapy has decreased diarrheal disease mortality worldwide but still accounts for the deaths of nearly 1.3 million children annually.^5-7^ In contrast, in developed nations mortality occurs in <1% of all hospitalized cases.^2,7^ We present the case of a toddler who succumbed to a diarrheal illness of unclear etiology. Although indolent at first, he declined precipitously in the pediatric intensive care unit (PICU). We describe our diagnostic dilemma in the management of his illness.

## Case Presentation

The 13-month-old ex-37-week-old male presented with a chief complaint of diarrhea. Stools were initially loose and watery, occurring 7 to 10 times per day. Associated symptoms included tenesmus that resolved following bowel movements. There was no history of fevers or emesis. Within 72 hours of symptom onset, the child was having frankly bloody stools.

He was primarily breast-fed until 12 months of age when cow’s milk–based formula was introduced. There was no history of exposure to unpasteurized milk or raw meat. Immunizations were up-to-date and developmental milestones appropriate for age. Past medical history was significant only for eczema. He had a family history of eczema, asthma, and lactose intolerance. There was no history of travel. The family owned no pets. The patient did attend a daycare where 3 other children recently had diarrheal illnesses.

After the onset of bloody stools, an evaluation for infection was undertaken, and the child was switched to a soy-based formula. Despite the change in formula, symptoms progressed with decreased activity and lethargy. Immediately prior to passing a stool, the patient would pull his legs up, grunt, and look very uncomfortable. On the sixth day of illness, he presented to the emergency department after passing gelatinous, bloody stools.

Presenting vital signs included a rectal temperature of 101°F, heart rate of 140/min, blood pressure 101/57 mm Hg, respiratory rate of 28/min, and oxygen saturation of 100% on room air. Initial complete blood count and complete metabolic panel were normal; albumin was 2.9 g/dL. A urinalysis showed ketonuria, proteinuria, and hematuria. Blood, urine, and stool cultures were sent (stool cultures were reportedly negative from the pediatrician’s office). C-reactive protein was elevated at 12.5 mg/L.

On examination, the child was tired-appearing, intermittently crying, and drawing legs up to his chest. He passed a frankly bloody stool and appeared more comfortable thereafter. Mucus membranes were dry. His abdomen was tender to palpation; bowel sounds were hypoactive. There was no active bleeding per rectum, anal fissures, or hemorrhoids.

Abdominal radiographs demonstrated a nonobstructive bowel gas pattern and increased density in the right lower quadrant suggestive of fluid collection. Ultrasound (US) demonstrated a significant amount of peritoneal fluid with marked rectosigmoid submucosal edema and bowel wall thickening and no evidence of either intussusception or appendicitis.

After fluid resuscitation, the child was admitted to the pediatric ward. During the first 6 hours of hospitalization, he had 8 loose, bloody stools, more generalized abdominal tenderness, and the appearance of red, beefy mucosa protruding from the anal sphincter; per the surgical team, the anal mucosa was edematous and protruded with patient’s Valsalva. Over the next 24 hours, clinical improvement was noted, with increased oral intake and decreased stools.

However, on hospital day 3, he suffered an acute decline. He was tachycardic (205/min) and hypotensive (44/28 mm Hg); cap refill was 2 to 3 seconds with intact peripheral pulses. He was in significant respiratory distress with tachypnea (47/min) and grunting; saturations on room air were 100%. His abdomen was significantly more distended and tender to palpation without rigidity.

Laboratory evaluation revealed leukocytosis (25 600 cells/µL) with a high percentage of immature forms (bands = 52%) and thrombocytosis (626 000 cells/µL). His basic metabolic panel showed hyponatremia (130 mmol/L) with a non–anion gap metabolic acidosis (bicarbonate 18 mmol/L, anion gap 9). Liver function panel was normal, but serum albumin level was 1.2 g/dL, glucose was 194 mg/dL, and international normalization ratio was 1.3. Blood urea nitrogen and creatinine were normal. Of note, stool studies from the emergency department were negative to date.

After transfer to the PICU, the patient received additional fluid resuscitation with 5% albumin. He was started on intravenous pipercillin/tazobactam at 10 mg/kg every 8 hours and metronidazole 8 mg/kg every 6 hours. Abdominal plain films demonstrated centralization of bowel loops, absence of gas in the rectum, dilated stomach, small bowel, and sigmoid colon, and bilateral pleural effusions. US revealed a large volume of ascites throughout the abdomen; multiple bowel loops with thickening, most notably in the left lower quadrant; edema; and diffuse thickening of the rectal wall with preserved blood flow. Abdominal computed tomography scan confirmed the presence of large volume ascites in the abdomen and pelvis with patchy areas of decreased mucosal enhancement, mucosal edema, and bowel wall thickening of the rectosigmoid colon.

Given our current suspicion for a secondary infectious abdominal process and his respiratory distress, abdominal paracentesis was performed. Fluid was turbid with 1600 nucleated cells (69% neutrophils, 12% lymphocytes, 18% monocytes), 1700 red blood cells, lactate dehydrogenase 177 U/L and albumin 1.2 g/dL. Over a 2-hour period, 1 L of fluid was drained and hemodynamic status remained well compensated.

During the first 6 hours of admission to the PICU, the child was visibly more comfortable, especially after paracentesis. His heart rate dropped to 150/min and systolic blood pressures stabilized between 90 and 110 mm Hg. He was on nasal cannula oxygen. His urine output averaged 1.3 mL/kg/h. Unfortunately, over the subsequent 6 hours, his clinical status declined. Tachycardia persisted despite administration of intravenous fluids. Noninvasive positive pressure ventilation was initiated for on-going respiratory distress. Surgery was reconsulted for potential exploration but deemed him unstable for the procedure. Approximately 12 hours after PICU admission, the child experienced complete cardiovascular collapse and died despite protracted cardiopulmonary resuscitative efforts.

**Figure 1.**
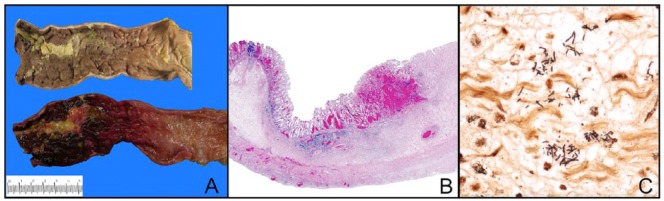
(A) Severe acute proctocolitis of rectosigmoid colon (top-mucosal surface, formalin-fixed; bottom-mucosal surface, unfixed; left-distal; right-proximal). There is severe acute inflammation, ulceration, hemorrhage, and necrosis of the rectosigmoid colon (left side of the figure) with gradual transition to relatively normal colon (right side of the figure). (B) Histologic section of the distal sigmoid colon shows acute necrosis involving the entire thickness of the bowel wall (top-mucosal surface; bottom-serosal surface, hematoxylin-eosin stain). (C) Gram negative bacilli in the edematous submucosa of the sigmoid colon (Gram stain).

At autopsy, the major finding was severe proctocolitis extending from the distal sigmoid colon to the anal verge (Figure 1A). The gastrointestinal tract was otherwise normal, that is, no volvulus, intussusception, obstruction, rupture, thrombosis, or appendicitis. There was no intracranial hemorrhage, pulmonary embolism, or myocarditis.

Histology of distal sigmoid and rectum revealed acute hemorrhagic mucosal necrosis with ulceration and marked submucosal edema (Figure 1B). In some areas, full thickness of the bowel wall was necrotic. Necrotic mucosa and the ulcer bed contained gram-negative bacilli, extending into the submucosa (Figure 1C). Culture of the sigmoid mesentery/mesenteric lymph node revealed lactose-fermenting gram-negative rods and enterococcus.

## Discussion

Worldwide infectious diseases, most notably diarrheal illnesses, represent the greatest cause of mortality in children less than 5 years of age.^7^ In the United States, mortality for diarrheal illnesses is less than 1%.^2,7^ Here, we present a case of a toddler with a protracted diarrheal illness and cardiovascular collapse due to overwhelming gram-negative bacteremia. The underlying cause of compromised colon barrier function remains unknown.

Intussusception, a pediatric gastrointestinal emergency and the most common cause of small bowel obstruction, with an annual incidence of 0.2%, warrants consideration.^8^ Two thirds of patients with intussusception are less than 1 year of age. Most commonly, the ileum telescopes into the colon but variants do occur. Pathologic lead points, such as Meckel’s diverticulum, tumor, vascular malformation, or enlarged mesenteric lymph nodes, are found in 2% to 12% of cases.^9^ Classical symptoms include colicky abdominal pain, vomiting, palpable mass, and diarrhea. Left untreated, bowel obstruction with mesenteric vascular compromise ensues with eventual bowel wall necrosis characterized by currant jelly stools.^8,10^ Diagnosis can accurately be made by US (sensitivity 98% to 100%), allowing for prompt treatment.^8,11^

Transient intussusception is a rare diagnosis without consistently reliable presenting signs and symptoms or reliable radiographic criteria, and US sensitivity is only 84%.^11^ Predisposing factors include bowel wall edema, abnormal motility, adhesion, and infections, including rotavirus.^8,12^ Moreover, distinction between transient small bowel intussusception and ileo-colic intussusception is not firmly established.^10,11,13^

Our infant was in the classic age group and presented with clinical characteristics consistent with intussusception: acute, colicky abdominal pain, irritability, lethargy with intermittent symptomatic relief, and currant jelly stools. Although no pathologic lead points were demonstrated on autopsy, bowel wall edema, abnormal motility, or infection may have caused intussusception.^12^ Histologic review of the ischemic and necrotic colon was reminiscent of intussusception pattern. While transient small bowel intussusception without a lead point has been described in infants and children, no case of transient colonic intussusception has ever been documented, making this diagnosis particularly challenging.^10^

Food allergic enterocolitis is another, albeit less likely, consideration. Disorders that primarily affect infants include acute gastrointestinal hypersensitivity reactions; protein-induced enterocolitis, proctitis, or proctocolitis; and allergic eosinophilic gastroenteropathies. Infants with dietary protein–induced proctitis or proctocolitis generally present in the first few months of life during breastfeeding due to the secretion of maternally ingested proteins in breast milk. Infants are typically healthy and present with blood mixed with mucus in their stools. Cow milk proteins, and to a smaller degree soy protein, are the most common triggers.^14^ With dietary protein enterocolitis, infants present with diarrhea, emesis, lethargy, and failure to thrive. Although cow milk proteins are the most common cause, rice, oats, cereal grains, and poultry have also been implicated.^15^ Eosinophilic gastroenteropathies are much less common, may affect any part of the gastrointestinal tract, and are characterized by postprandial nausea, dysphagia, abdominal pain, emesis, and diarrhea. The allergic subset of this disorder is usually caused by milk, egg, wheat, and soy.^16^

Our infant not only had a personal and family history of atopy but also experienced diarrhea with both cow milk and soy protein ingestion. Histologic sections of the less necrotic areas of the distal sigmoid showed lymphoid nodular hyperplasia in the lamina propria, a heavy infiltrate of lymphocytes, macrophages, and eosinophils in the lower lamina propria and focally in the muscularis propria and sigmoid serosa, all consistent with the diagnosis of food protein–induced enterocolitis (FPIES).^17^

In the present case, the variable clinical exam, non-specific radiographic findings, and negative infectious studies compromised our ability to find a unifying diagnosis. The abdominal examination did not support a decision for an exploratory laparotomy. The onset of hematochezia with absent infectious or anatomic causes likely signaled compromise in bowel barrier function, from ischemia (intussusception) or ulceration (FPIES), which permitted enteric gram-negative bacterial invasion. Ensuing bacteremia and bowel necrosis led to cardiovascular collapse.

## Conclusion

Thus, in noninfectious diarrheal illnesses wherein the patient’s clinical symptoms progress despite a conservative approach, rare entities such as transient intussusception or food protein–induced enterocolitis syndromes are valid considerations. We further endorse early PICU admission and surgical involvement with exploratory laparotomy, especially in the presence of bacterial peritonitis and non-reassuring radiographic findings, such as absence of air in the rectum.
